# Relative Handgrip Strength Is a Simple Indicator of Cardiometabolic Risk among Middle-Aged and Older People: A Nationwide Population-Based Study in Taiwan

**DOI:** 10.1371/journal.pone.0160876

**Published:** 2016-08-25

**Authors:** Wei-Ju Lee, Li-Ning Peng, Shu-Ti Chiou, Liang-Kung Chen

**Affiliations:** 1 Aging and Health Research Center, National Yang Ming University, Taipei City, Taiwan; 2 Institute of Public Health, National Yang Ming University, Taipei City, Taiwan; 3 Department of Family Medicine, Taipei Veterans General Hospital Yuanshan Branch, Yilan County, Taiwan; 4 Center for Geriatrics and Gerontology, Taipei Veterans General Hospital, Taipei City, Taiwan; 5 Health Promotion Administration, Ministry of Health and Welfare, Taipei City, Taiwan; Medizinische Universitat Innsbruck, AUSTRIA

## Abstract

**Background:**

Muscle strength may play an important role in cardiovascular health. The study was intended to evaluate the association between cardiometabolic risk, risk of coronary artery disease and handgrip strength by using the relative handgrip strength.

**Materials and Methods:**

Data of 927 Taiwanese aged 53 years and older (510 men and 417 women) were retrieved from a nationwide representative population-based cohort cross-sectional study in 2006. All participants were interviewed face-to-face and received measures of anthropometry, dominant handgrip strength, relative handgrip strength (summation of both handgrip strength divided by body mass index) and serum biomarkers.

**Results:**

Multivariate linear regression analysis showed the significant association between relative handgrip strength and favorable cardiometabolic risk factors including blood pressure, triglyceride, total cholesterol to high density cholesterol(HDL-C) ratio, glycohemoglobin (HbA1c), uric acid, Framingham risk score in men, and HDL-C, fasting glucose, HbA1c, log hsCRP in women. Dominant hand grip strength was only associated with log hsCRP in women. (*p*<0.05 for all), but was not significant associated with all cardiovascular biomarkers and FRS in both sex.

**Conclusions:**

Joint with handgrip strength and body size, as relative handgrip strength, may be a better tool to capture conceptual concomitant health, which may be a simple, inexpensive, and easy-to-use tool when targeting cardiovascular health in public health level.

## Introduction

The importance of muscle fitness and physical performance to healthy aging had been reported extensively in recent years. A great number of epidemiological studies supported the association of muscle fitness and functional decline, frailty, metabolic syndrome, diabetes mellitus, mobility and mortality.[1–8] In the measurement of muscle fitness, handgrip strength was a simple, quick, reliable, and inexpensive tool, and was widely accepted method in the diagnostic algorithm of sarcopenia.[9–11] Handgrip strength represented the muscle strength of forearm and upper limb when measured by a sitting position, and also included lower limbs and core muscle strength when tested by a standing position.[12] In the diagnosis of sarcopenia, different muscle index (adjusted by weight or height) had been used and low muscle mass identified by different muscle index eventually differed greatly.[13] Recently, the Foundation for the National Institutes of Health (FNIH) Sarcopenia Project proposed a new approach for sarcopenia diagnosis, in which skeletal muscle mass was adjusted by body mass index instead of body height or weight.[14]

Several previous studies have shown the association between handgrip strength and cardiovascular risk.[4, 6, 15, 16] A large prospective cohort study of 154,000 individuals aged 35–70 years have identified the association between impaired of handgrip strength and cardiovascular mortality, but not incident diabetes mellitus during the follow-up for 4 years.[1] A cohort study of 2,677 residents aged 59–73 years of Herefordshire reported a significant inverse association between handgrip strength and metabolic syndrome, as well as insulin resistance.[4] Data from the National Health and Nutrition Examination Survey (NHANES) also supported aforementioned findings.[6] Some studies suggested that the association between cardiometabolic risk and handgrip strength only existed in women,[17] but results of another study supported otherwise.[18] On the other hand, the association between cardiovascular health and handgrip strength was insignificant in other studies.[19, 20] These confusing results may be caused by using the dominant handgrip strength with or without corrected with weight, which suggested the important role of body size in evaluating the association between muscle strength and cardiometabolic risk.

Using BMI for the adjustment of muscle strength has been recommended in the research of muscle health.[14, 21] Relative handgrip strength, defined by summation of both hands strength divided by BMI, served as an easy instrument to measure muscle health in public health and in clinical practice. Higher relative handgrip strength was associated with lower mobility limitation[21]. The association between muscle strength and cardiometabolic risk was usually done by using dominant handgrip strength,[16, 19, 22] which may result in contradictory results and the association has never been evaluated by using the relative handgrip strength. The main aim of this study was to examine the association between cardiovascular health and muscle health by using relative/dominant handgrip strength through a national representative cohort in Taiwan.

## Materials and Methods

### Participants and study design

Data from the second wave of Social Environment and Biomarkers of Aging Study (SEBAS) in 2006 was used for this study. The study sample was obtained by using a multistage proportional-to-size sampling strategies to produce a national representative sample of the Taiwanese aged 53 years and over. The aim of SEBAS was to explore the relationship between demographic characteristics, social environment, various measurements for health, incorporating biological markers and physical examinations. The whole study design, sampling strategies and cohort profile have been reported previously.[23] In brief, 1,284 of 1,659 participants invited for SEBAS in 2006 received an in-person home interview by well-trained research nurses after they were fully consented. Among them, 1,036 participants attended nearby hospitals for complete physical assessments and overnight fasting blood sampling. Data of 109 participants with incompleteness were excluded for analysis, which left data of 927 participants for analysis. The Institutional Review Boards at Princeton University, Georgetown University, and the Joint Institutional Review Board of Taiwan approved the whole study.

### Measurements

Anthropometric measurements of body height and weight were done by standard procedure. BMI was calculated as body weight in kilograms divided by body height in meter square. Isometric handgrip strength was measured by a North Coast^™^ hydraulic hand dynamometer (NC70142) in a sitting position by holding the dynamometer at a 90-degree flexion of their elbow. Subjects with the following conditions were excluded for handgrip strength measurements. First, subjects had recent injury or worsening of pain of the wrist or hand. Second, subjects had current swelling/inflammation or severe pain in their wrist or hands. Last, participants received surgery of the wrist or hands within recent three months. Interviewers would adjust the dynamometer to fit individual’s palm size. Maximal readings of three measurements from both hands were recorded. Absolute handgrip strength was summed from readings of both hands. Relative hand grip strength was defined as absolute handgrip strength divided by BMI [21]. Walking speed was measured with a 3-meter walking evaluation with a static start.

Serial biomarkers related to cardiovascular health were measured in this study, including systolic blood pressure, diastolic blood pressure, fasting glucose, glycolated hemoglobin A1c (HbA1c), total cholesterol, triglyceride, and high density lipoprotein cholesterol (HDL-C)[24], high sensitivity C reactive protein (hsCRP)[25], and uric acid[26]. Blood pressure was taken by an automatic monitor (Omron^®^ Model HEM-7011) three times (with maximum five attempts) successively with at least one-minute interval between each reading. The average of the three blood pressure measurements was used for analysis. All those serum biomarkers were collected from overnight fasting participants. Favorable cardiovascular outcomes were defined as lower blood pressure, lower total cholesterol, lower triglyceride, higher HDL-C, lower cholesterol to HDL-C ratio, lower uric acid, and lower hsCRP as previously reported. [6] These cardiovascular outcomes were used as continuous variable for the interpretation of their association with handgrip strength. Meanwhile, the Framingham Risk Score was used as the measurement to estimate the risk of cardiovascular disease.[24] Metabolic syndrome defined by using National Cholesterol Education Program (NCEP) Adult Treatment Panel III (ATP III) guidelines[27] and the International Diabetes Federation (IDF) were used to surrogate the overall cardiometabolic health as well[28]. Charlson comorbidity Index (CCI) was used to measure conditions of comorbidity [29].

Since the use of statins may be associated with muscle pain and reduced muscle strength, it was entered in the regression model as a covariates [30]. Other covariates such as age, and exercises, were adjusted in the analytical models due to the potential influences on muscle strength. All participants were asked for bring all their medications to interviewers to determine whether use of any lipid lowering medication or not. Participants who have a habit of exercise three times or more per week would be classed as having regular exercise.

### Statistical analysis

The continuous variables was expressed as mean ± standard deviation, and categorized variables was expressed as number (%). An independent student t test was used to compare gender differences in numerical variables. Chi-square test was used to determine the association between gender and metabolic syndrome. Due to the significant gender differences in handgrip strength, data of women and men were separated for further analysis in this study. All biomarkers of cardiovascular health and Framingham Risk Score (FRS) were tested by normal quantile plot of their residues. Variables as hsCRP did not meet the assumption of normality in linear regression, and were log transformed for analysis. Because different measure units existed in relative handgrip strength and dominant/absolute handgrip strength, both measurements were standardized by their interquartile range to assist the interpretation of regression coefficient [31]. The magnitude effect of observational association has absolutely no change after rescaling units. Rescaling by interquartile instead of standard deviation have an attractive intuitive interpretation and was adopted in the current analytic strategy. When interpreting results of regression analysis, it represents difference between those with “typical high” value, i.e. those in the middle of the upper half of the distribution (seventy-five percentile), and those with typical low value, i.e. those in the middle of the lower half of the distribution (twenty-five percentile) [32].

Multivariate logistic regression was used to examine the association between relative handgrip strength, dominant handgrip strength and metabolic syndrome. Pearson correlation was used to examine the correlation between relative/dominant handgrip strength and BMI in women and men.

Youden’s index[33], a main summary statistic from the receiver operating characteristic curve (ROC) analysis and calculated as sensitivity plus specificity minus 1, was used to determine the optimal cut-off values to achieve the greatest effectiveness of the relative handgrip strength for prediction of metabolic syndrome. A p-value (2-tailed) less than 0.05 was considered statistically significant. All analyses were carried out with the SAS statistical package, version 9.4 (SAS Institute, Inc., Cary, NC).

## Results

Overall, data of 927 subjects (mean age: 65.6±9.4 years) were used for study and 6.8% of them reported using statins currently. Both women and men had the similar proportion of using statins. Dominant handgrip strength ranged from 2.0 to 42.0 kg with the interquartile range (IQR) of 16.0–24.0 kg in women and 6.0 to 68.0 kg with IQR of 28.0–40.0 kg in men. Relative handgrip strength ranged from 0.08 to 3.29 m^2^ with IQR 1.25–1.87 m^2^ in women and 0.57 to 5.69 m^2^ with IQR 2.22–3.15 m^2^ in men, respectively. Compared to women, men were older, having higher dominant and relative handgrip strength, less favorable cardiovascular outcomes (higher diastolic blood pressure, lower HDL-C, higher uric acid, higher cholesterol to HDL-C ratio, and higher FRS), but similar comorbidity and proportion of metabolic syndrome (Table 1).

**Table 1:** Clinical characteristics, biomarkers and metabolic syndromes of SEBAS by sex.

	Men		Women		
	Mean	STD	Mean	STD	*p* value
Total number	510		417		
Age(years)	66.3	9.5	64.6	9.1	**0.005**
Dominant Handgrip strength(kg)	33.7	8.7	20.0	6.1	**<0.001**
Absolute Handgrip strength (kg)	65.8	16.7	38.4	11.9	**<0.001**
Relative Handgrip strength (kg/BMI)	2.7	0.7	1.6	0.5	**<0.001**
Body mass index(kg/m^2^)	24.6	3.2	24.9	3.7	0.184
Blood pressure (mmHg)					
Systolic	140.2	19.9	138.0	20.8	0.104
Diastolic	80.4	12.1	77.4	10.8	**<0.001**
Pulse pressure	59.8	14.5	60.6	15.6	0.400
Fasting glucose(mg/dl)	107.6	31.2	106.0	30.5	0.436
Cholesterol(mg/dl)					
Total cholesterol	192.3	37.0	206.4	37.2	**<0.001**
Triglyceride	113.8	72.3	112.5	66.2	0.775
HDL cholesterol	44.8	12.9	51.7	14.2	**<0.001**
Total cholesterol to HDL cholesterol ratio	4.5	1.2	4.2	1.1	**<0.001**
HbA1c(%)	6.1	1.3	6.2	1.2	0.853
HsCRP(mg/dl)	0.3	0.7	0.2	0.5	0.255
Uric acid(mg/dl)	6.3	1.4	5.4	1.3	**<0.001**
Walking speed(m/s)	0.9	0.3	0.8	0.3	**<0.001**
Charlson Comorbidity Score	0.8	1.0	0.7	1.0	0.425
Framingham Risk Score	17.4	7.6	6.2	6.5	**<0.001**
Metabolic syndromes					
IDF definition, n, (%)	141	(27.6)	133	(32.0)	0.152
NCEP ATPIII, n(%)	184	(36.1)	155	(37.3)	0.711

Values of absolute hand grip strength were calculated by summation of dominant and non-dominant hand grip strength. Values of relative handgrip strength were calculated from absolute handgrip strength divided by body mass index.

In both genders, relative handgrip strength was significantly associated with favorable outcomes of HDL-C. In men, relative handgrip strength was associated with lower systolic blood pressure, lower triglyceride, lower cholesterol to HDL-C ratio, lower uric acid and lower FRS. However, relative handgrip strength was associated with lower fasting glucose, lower HbA1c and lower log hsCRP in women. These associations would be insignificant in men when using dominant handgrip strength with adjustment for BMI. In women, the associations between dominant handgrip strength and favorable outcomes was only significant in log hsCRP (Table 2). Association between absolute handgrip strength, i.e. summation of non-dominant and dominant hand grip strength, and cardiovascular outcomes were similar with that with dominant handgrip strength (Data not shown).

**Table 2:** Results of multiple linear regression of relative hand grip strength (strength/BMI) and dominant hand grip strength on cardiovascular biomarkers.

	Relative hand grip strength per IQR^†^				Dominant hand grip strength per IQR^‡^			
	Men		Women		Men		Women	
	Estimate(SE)	*p*	Estimate(SE)	*p*	Estimate(SE)	p	Estimate(SE)	*p*
Systolic blood pressure	-2.55(1.31)	**0.051**	-1.34(1.41)	0.340	0.04(1.42)	0.977	0.57(1.49)	0.704
Diastolic blood pressure	-1.31(0.78)	0.092	-0.16(0.78)	0.832	-0.13(0.85)	0.879	1.01(0.82)	0.219
Pulse pressure	-1.24(0.86)	0.152	-1.18(0.98)	0.228	0.17(0.96)	0.858	-0.45(1.04)	0.668
Total cholesterol	-4.37(2.45)	0.075	3.35(2.65)	0.207	-1.38(2.74)	0.615	4.99(2.82)	0.078
Triglyceride	-12.89(4.69)	**0.006**	-5.53(4.68)	0.238	-0.58(5.09)	0.910	-4.34(4.96)	0.383
HDL cholesterol	1.88(0.85)	**0.028**	1.98(1.01)	**0.051**	-0.68(0.92)	0.459	0.76(1.07)	0.475
Total cholesterol to HDL cholesterol	-0.27(0.08)	**0.001**	-0.03(0.08)	0.741	0.03(0.09)	0.760	0.08(0.08)	0.313
Fasting glucose	-1.51(2.05)	0.462	-4.96(2.05)	**0.016**	-0.90(2.30)	0.695	-3.00(2.17)	0.167
HbA1c	-0.12(0.08)	0.153	-0.25(0.08)	**0.001**	-0.13(0.09)	0.177	-0.15(0.08)	0.072
Uric acid	-0.30(0.09)	**0.002**	-0.11(0.09)	0.224	-0.09(0.10)	0.360	0.03(0.09)	0.778
log hsCRP	-0.09(0.05)	0.093	-0.18(0.05)	**<0.001**	-0.11(0.06)	0.063	-0.15(0.05)	**0.005**
Framingham Risk Score	-1.56(0.44)	**<0.001**	-0.36(0.30)	0.231	-0.44(0.48)	0.352	-0.07(0.32)	0.822

SE denotes standard error. log hsCRP denotes values of high sensitive C reactive protein was logarithm transformed. HbA1c denotes glycolated hemoglobin, IQR denotes interquartile range; IQR for dominant handgrip strength was 8.0 kg in women and 12.0 kg in men. IQR for relative handgrip strength was 0.62 m^2^ in women and 0.92 in men.

^†^Adjusted for age, exercise, Charlson comorbidity index, use of statin and walking speed

^‡^Adjusted for age, exercise, Charlson comorbidity index, use of statin, body mass index and walking speed

**Fig 1:**
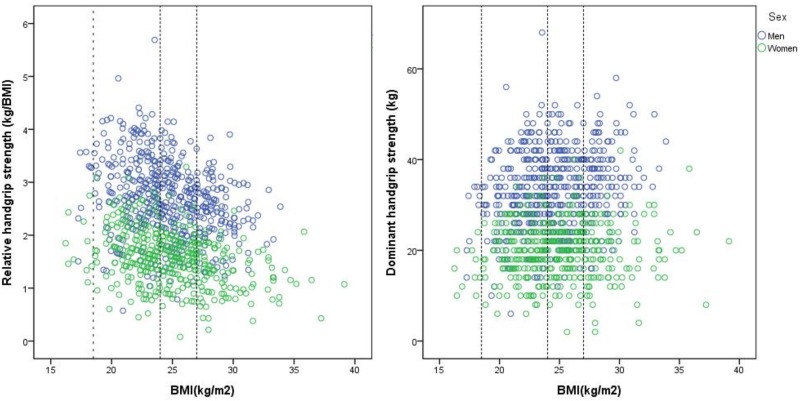
Relative grip strength and dominant grip strength versus body mass index by gender in Social Environment and Biomarkers of Ageing Study.

Table 3 summarized the results of multivariate logistic regression that higher relative handgrip strength per IQR change was associated with lower odds of having metabolic syndrome of NCEP ATPIII and IDF in both women and men. For men, risk of having metabolic syndrome defined by NCEP ATPIII would be lower by 54% with the increase of each interquartile relative handgrip strength. It also suggested that those with relative handgrip strength of 75th percentile had 54% lower risk of having metabolic syndrome than those with relative handgrip strengths of 25th percentile. These associations became insignificant when using dominant handgrip strength with adjustment for BMI. The relative handgrip strength was inversely correlated with BMI in both women (r = - 0.35, p<0.001) and men (r = -0.27, p<0.001). The dominant handgrip strength was correlated with BMI only in men (r = 0.20, P<0.001), but not in women (r = 0.08, p = 0.096). (Fig 1)

**Table 3:** Results of multivariable logistic regression of gender specific handgrip strength on metabolic syndromes.

	Men				Women			
	OR	95%CI		*p*	OR	95%CI		*p*
Relative handgrip strength per IQR change^†^								
ATPIII MetS	0.46	0.32	0.65	<0.001	0.64	0.45	0.92	0.015
IDF MetS	0.40	0.28	0.57	<0.001	0.54	0.38	0.79	0.001
Dominant handgrip strength per IQR change ^‡^								
ATPIII MetS	0.98	0.95	1.01	0.187	1.01	0.69	1.50	0.949
IDF MetS	0.98	0.95	1.01	0.256	0.99	0.66	1.49	0.964

IQR denotes interquartile range. ATPIII MetS denotes metabolic syndrome defined by Third Report of the Expert Panel on Detection, Evaluation, and Treatment of High Blood Cholesterol in Adults; IDF Mets denotes the International Diabetes Federation (IDF) Worldwide Definition of the Metabolic Syndrome; IQR for dominant handgrip strength was 8.0 kg in women and 12.0 kg in men. IQR for relative handgrip strength was 0.62 m^2^ in women and 0.92 in men.

^†^Adjusted for age, exercise, Charlson comorbidity index, use of statin and walking speed

^‡^Adjusted for age, exercise, Charlson comorbidity index, use of statin,body mass index and walking speed

The cut-off values of relative hand grip strength, determined by ROC analysis, for prediction of metabolic syndrome defined by NCEP were 2.91 m^2^ (sensitivity 0.78, specificity 0.45, c statistic 0.633) in men and 1.51 m^2^ (sensitivity 0.60, specificity 0.65, c statistic 0.648) in women; for prediction of metabolic syndrome defined by IDF were 3.00 m^2^ (sensitivity 0.87, specificity 0.40, c statistic 0.656) in men and 1.51 m^2^ (sensitivity 0.66, specificity 0.66, c statistic 0.686) in women.

## Discussion

This study examined the association between relative handgrip strength and cardiometabolic risk, FRS, and metabolic syndrome. The principal finding was that relative handgrip strength was significantly associated with favorable cardiometabolic risk, including blood pressure, triglyceride, HDL-C, total cholesterol to HDL-C ratio, uric acid, FRS and metabolic syndrome in men, and HDL-C, fasting glucose, HbA1c, log hsCRP and metabolic syndrome in women. All these associations of dominant hand grip strength and biomarkers were insignificant except association between log hsCRP and dominant grip strength in women. Findings of the present study were consistent with previously studies [6, 22]. Moreover, it implied that relative handgrip strength, but not dominant handgrip strength, was a more appropriate instrument for evaluation of cardiovascular risk profile. Some previous studies failed to demonstrate the association between cardiovascular risk and handgrip strength, which may be resulted from using dominant handgrip strength instead of relative handgrip strength [17–20]. A study of 647 older people in Korea found that weight-adjusted handgrip strength was associated with cardiovascular risk in men only, and the effect became weaker among people aged 75 years and older[18]. However, this association was observed in a Japanese cohort by using absolute handgrip strength nor in a rural Taiwanese cohort by using sarcopenia defined by the European Working Group on Sarcopenia in Older People (EWGSOP) [17, 19]. A study of older Japanese in the rural area using weight-adjusted handgrip strength showed the association between lower cardiometabolic risk and higher handgrip strength [34]. Results from this study suggested that relative handgrip strength, instead of dominant/absolute handgrip strength, was significantly associated with metabolic syndrome. Although cut-off values were proposed by ROC analysis in the study, the sensitivity to identify metabolic syndrome in women was low, as was the specificity to identify metabolic syndrome in men. Fowles and Lawman et al. argued that handgrip strength *per se* may be inadequately addressing the issue of body size when targeting in cardiovascular health [20]. Relative handgrip strength treated body size and grip strength simultaneously, which may be the plausibility why relative handgrip strength was superior to dominant grip strength in cardiovascular health.

FRS was a scoring system made by traditional cardiovascular risk factors to predict the ten-year probability of coronary heart disease, which was considered a suitable tool for Asia population but may be overestimated cardiovascular risk of women among Taiwanese [35]. Results of this study showed insignificant association between dominant handgrip strength and FRS, which was in line with the previous report [36]. Due to the age limitation of Framingham Heart study participants, some argued that FRS was not suitable for people aged 85 and over, and hsCRP would be a better indicator of cardiovascular health instead[37]. A study of 2,193 older people with 8-year follow-up suggested that FRS would underestimate the risk of coronary heart disease, especially in women, despite that traditional risk factors (such as total cholesterol, total cholesterol to HDL-C ratio etc.) were still the best predictors [38]. Therefore, it may be plausible that relative handgrip strength was associated FRS in men but not in women, although relative handgrip strength was associated with hsCRP in both genders.

Pulse pressure has been reported as an indicator for arterial stiffness. In the present study, handgrip strength was not associated with pulse pressure, which was consistent with previous studies [19, 39]. A recent study of 1,467 participants with normal weight from the 2011–2012 National Health and Nutrition Examination Survey showed that lower handgrip strength was associated with risk of diabetes mellitus and hypertension, and suggested using the relative handgrip strength as a public screening tool [40]. In this study sample, relative handgrip strength was associated with blood pressure in men and glucose/HbA1c in women. Lower handgrip strength for women compared to men started from early adulthood may be potential explanation for gender differences [17].

The previous study has shown that muscle mass indices adjusted by either height or weight would identify subjects with low muscle mass in very different body size [13]. Foundation of National Institute of Health (FNIH) proposed to capture body weight and body height in a single tool by using body mass index [14]. Similar concepts may be applied to handgrip strength measurement, Choquette et al., suggested to use BMI-adjusted handgrip strength as a new screening instrument for population at risk of mobility limitation[21]. The sensitive and specificity of using BMI-adjusted handgrip strength to associate with the risk of mobility limitation were 75% and 64% in men and 67% and 59% in women in a Chinese cohort sample [2]. Results of current study extended the utilization of this new tool, relative handgrip strength, from mobility limitation to cardiovascular health.

Despite all efforts went into this study, there were some limitations. First, due to cross-sectional study design, the causal relationship between muscle strength and cardiovascular health may not be clarified. Second, the current study did not measure the quantity of muscle mass, so the effect of muscle quantity on the muscle strength may be overlooked. Third, the diagnosis of previous cardiovascular disease was self-reported by the participants to the interviewing research nurses.

## Conclusion

Relative handgrip strength was significantly associated between cardiometabolic risk, and the association was stronger than that using dominant handgrip strength. Relative handgrip strength is a simple, convenient and inexpensive tool when targeting in cardiovascular health in a community setting or daily clinical practice.
